# Identifying thresholds for classifying moderate-to-heavy soil-transmitted helminth intensity infections for FECPAK^G2^, McMaster, Mini-FLOTAC and qPCR

**DOI:** 10.1371/journal.pntd.0008296

**Published:** 2020-07-02

**Authors:** Bruno Levecke, Piet Cools, Marco Albonico, Shaali Ame, Cécile Angebault, Mio Ayana, Jerzy M. Behnke, Jeffrey M. Bethony, Giuseppe Cringoli, Daniel Dana, Bertrand Guillard, Nguyen Thi Viet Hoa, Gagandeep Kang, Deepthi Kattula, Jennifer Keiser, Andrew C. Kotze, Leonardo F. Matoso, Maria P. Maurelli, James S. McCarthy, Zeleke Mekonnen, Greg Mirams, Antonio Montresor, Rodrigo Corrêa Oliveira, Maria V. Periago, Simone A. Pinto, Laura Rinaldi, Somphou Sayasone, Laurentine Sumo, Louis-Albert Tchuem-Tchuenté, Dang Thi Cam Thach, Eurion Thomas, Ahmed Zeynudin, Jaco J. Verweij, Johnny Vlaminck, Jozef Vercruysse

**Affiliations:** 1 Department of Virology, Parasitology and Immunology, Ghent University, Merelbeke, Belgium; 2 Center for Tropical Diseases, Sacro Cuore Don Calabria Hospital, Negrar, Italy; 3 Department of Life Sciences and Systems Biology, University of Turin, Turin, Italy; 4 Public Health Laboratory-Ivo de Carneri, Chake Chake, United Republic of Tanzania; 5 Clinical Laboratory, Institut Pasteur, Phnom Penh, Cambodia; 6 Jimma University Institute of Health, Jimma University, Jimma, Ethiopia; 7 School of Life Sciences, University of Nottingham, Nottingham, United Kingdom; 8 Microbiology, Immunology, and Tropical Medicine, George Washington University Medical Center, Washington D.C., United States of America; 9 Department of Veterinary Medicine and Animal Production, University of Naples Federico II, Naples, Italy; 10 Department of Parasitology, National Institute of Malariology, Parasitology and Entomology, Ha Noi, Vietnam; 11 Department of Gastrointestinal Sciences, Christian Medical College, Vellore, India; 12 Department of Medical Parasitology and Infection Biology, Swiss Tropical and Public Health Institute, Basel, Switzerland & University of Basel, Basel, Switzerland; 13 Division of Agriculture and Food, Commonwealth Scientific and Industrial Research Organization, St. Lucia, Australia; 14 Laboratory of Molecular and Cellular Immunology, Research Center René Rachou - FIOCRUZ, Belo Horizonte, Brazil; 15 QIMR Berghofer Medical Research Institute, University of Queensland, Brisbane, Australia; 16 Techion Group Ltd, Dunedin, New Zealand; 17 Department of Control of Neglected Tropical Diseases, World Health Organization, Geneva, Switzerland; 18 Lao Tropical and Public Health Institute, Ministry of Health, Vientiane Capital, Lao People’s Democratic Republic; 19 Centre for Schistosomiasis and Parasitology, University of Yaoundé I, Yaoundé, Cameroon; 20 Techion Group Ltd, Aberystwyth, United Kingdom; 21 Laboratory for Medical Microbiology and Immunology, Elisabeth-Tweesteden Hospital, Tilburg, The Netherlands; Emory University, UNITED STATES

## Abstract

The World Health Organization (WHO) has defined moderate-to-heavy intensity (M&HI) infections with soil-transmitted helminths (*Ascaris lumbricoides*, *Trichuris trichiura* and the two hookworms, *Ancylostoma duodenale* and *Necator americanus*) based on specific values of eggs per gram of stool, as measured by the Kato-Katz method. There are a variety of novel microscopy and DNA-based methods but it remains unclear whether applying current WHO thresholds on to these methods allows for a reliable classification of M&HI infections. We evaluated both WHO and method-specific thresholds for classifying the M&HI infections for novel microscopic (FECPAK^G2^, McMaster and Mini-FLOTAC) and DNA-based (qPCR) diagnostic methods. For this, we determined method-specific thresholds that best classified M&HI infections (defined by Kato-Katz and WHO thresholds; reference method) in two multi-country drug efficacy studies. Subsequently, we verified whether applying these method-specific thresholds improved the agreement in classifying M&HI infections compared to the reference method. When we applied the WHO thresholds, the new microscopic methods mainly misclassified M&HI as low intensity, and to a lesser extent low intensity infection as M&HI. For FECPAK^G2^, applying the method-specific thresholds significantly improved the agreement for *Ascaris* (moderate → substantial), *Trichuris* and hookworms (fair → moderate). For Mini-FLOTAC, a significantly improved agreement was observed for hookworms only (fair → moderate). For the other STHs, the agreement was almost perfect and remained unchanged. For McMaster, the method-specific thresholds revealed a fair to a substantial agreement but did not significantly improve the agreement. For qPCR, the method-specific thresholds based on genome equivalents per ml of DNA moderately agreed with the reference method for hookworm and *Trichuris* infections. For *Ascaris*, there was a substantial agreement. We defined method-specific thresholds that improved the classification of M&HI infections. Validation studies are required before they can be recommended for general use in assessing M&HI infections in programmatic settings.

## Author summary

The prevalence of moderate-to-heavy intensity (M&HI) infections is a key indicator for measuring the success of large-scale deworming programs for intestinal worms because they account for the majority of the worm-attributable morbidity. Currently, intestinal worm infections are classified as M&HI when the number of worm eggs that are microscopically detected in stool using a standard diagnostic method exceeds a threshold set by the World Health Organization. Over the years, a variety of new promising diagnostic methods have been introduced for the diagnosis of intestinal worms. Although they have some important advantages over the current standard method, it is not clear whether they can reliably classify M&HI infections. This is because their test results either systematically indicate lower egg counts or are expressed in a unit other than eggs per gram of stool (e.g, concentration of worm DNA), warranting the need for method-specific thresholds. We defined method-specific thresholds and verified whether they increased the correct classification of M&HI infections. Overall, our results indicate that method-specific thresholds improved the classification of M&HI infections, but that further validation is required before they can be recommended for evaluating the occurrence M&HI infections in large-scale deworming programs.

## Introduction

Soil-transmitted helminths (STHs, *Ascaris lumbricoides* (*Ascaris*), *Trichuris trichiura* (*Trichuris*) and the hookworms, *Necator americanus* (*Necator*) and *Ancylostoma duodenale* (*Ancylostoma*) affect one quarter of the world population, and are responsible for the loss of more than three million disability-adjusted life years (DALYs) [1,2]. To combat STHs, the World Health Organization (WHO) and national health authorities aim to eliminate STHs as a public health problem by means of large-scale deworming programs [3,4]. WHO defines soil-transmitted helminthiases as a public health problem if at least 1% of school-aged children have moderate-to-heavy intensity (M&HI) STH infections (most of the morbidity due to STHs is caused by infections of M&HI infections) [4]. Since the 1990s, Kato-Katz has been the WHO recommended diagnostic method for quantifying eggs in stools, and for subsequently classifying the intensity of infections based on the obtained fecal egg counts (FECs; expressed as eggs per gram of stool (EPG)) into light, moderate and heavy (see Table 1; [5,6]). During the last decade, a variety of new diagnostic methods have been introduced to the STH-field, including both microscopy-based (e.g., FECPAK^G2^ [7,8], McMaster [9] and (Mini-)FLOTAC [10,11], and DNA-based methods (qPCR; [12–13])). Each of these methods have important advantages and disadvantages over the Kato-Katz. Important advantages are a clearer microscopic view (FECPAK^G2^, McMaster and (Mini-)FLOTAC) [14–16], a higher clinical sensitivity (= proportion of diseased individuals correctly diagnosed as infected; (Mini-)FLOTAC and qPCR) [17–19], opportunities for automated egg counting and quality control (FECPAK^G2^) [20], and the abilities to differentiate hookworm species and to simultaneously detect parasites other than STHs (qPCR) [21–23]. Chief limitations of these novel methods are the need for well-equipped laboratories with well-trained technicians (FLOTAC; qPCR), and the higher cost of processing large numbers of samples (FECPAK^G2^; McMaster, Mini-FLOTAC and qPCR). In addition, it is not clear whether these methods can be employed to monitor progress towards the program goals. For example, it has been shown that these new microscopy-based methods often result in significantly lower FECs compared to Kato-Katz, which may lead to an underestimation of the proportion of M&HI infections. As a result of this, incorrect and premature programmatic decision could be made that soil-transmitted helminthiases have been eliminated as a public health problem [8,9,19]. Moreover, in the case of DNA-based methods, there is a difference in the analytical unit (EPG for Kato-Katz and units reflecting DNA concentration for qPCR). This difference in analytical units is further complicated by the absence of single universally agreed unit for estimating DNA concentration in stools [19,23], which impedes setting thresholds that can be globally applied. A unit that theoretically holds most promise as a universal unit is the number of genome equivalents / ml DNA extract (GE/ml). Recently, Cools et al. (2019) [19] indicated that DNA concentration expressed as GE/ml correlated with FECs reported by Kato-Katz, highlighting that implementation of qPCR-specific criteria for M&HI infections is possible.

**Table 1:** Classes of soil-transmitted helminth infection intensities based on Kato-Katz fecal egg counts. The fecal egg counts are expressed in eggs per gram of stool. The thresholds were introduced by the World Health Organization in 1987 and 1998 [5,6].

	Light	Moderate	Heavy
*Ascaris*	1–4,999	5,000–49,999	≥50,000
*Trichuris*	1–999	1,000–9,999	≥10,000
Hookworm	1–1,999	2,000–3,999	≥4,000

In the present study, we determined whether the current operational WHO thresholds defining M&HI infections can be applied for FECPAK^G2^, McMaster, Mini-FLOTAC and qPCR. For the microscopic methods, we also verified whether these method-specific thresholds improved the agreement in classifying M&HI infections compared to the reference method (Kato-Katz and WHO thresholds).

## Methods

### Ethics statement

This study analyzed data obtained during two multi-country studies designed to assess the efficacy of a single oral dose of albendazole against STH infections in school-aged children. The first study was performed in Ethiopia, Lao PDR and Pemba Island (Tanzania) [19,24,25]. The overall protocol for this study was approved by the Ethics Committee of the Faculty of Medicine and Health Sciences, Ghent University (Ref. No B670201627755). Subsequently, approval was obtained by the Ethical Review Boards associated with each trial site (Ethical Review Board of Jimma University, Jimma, Ethiopia: Ref. No RPGC/547/2016; National Ethics Committee for Health Research, Vientiane Capital, Lao PDR: Ref. No 018/NECHR; Zanzibar Health Research Council, United Republic of Tanzania: Ref. No ZAMREC/0002/February/2015). Parent(s)/guardians of participants in this study were asked to sign an informed consent document indicating that they understood both the purpose of the procedures required for the study, and that they were willing to let their child participate in the study. If the child was 6–12 years, he or she had to orally agree in order to participate in the study. Participants over the age of 12 were only included if they signed an informed consent document indicating that they understood both the purpose of the study and procedures required for the study, and that they were willing to participate in the study. This study is registered under the ClinicalTrials.gov identifier NCT03465488.

The second study was conducted in Brazil, Cameroon, India, Pemba Island (Tanzania) and Vietnam [9,26]. The overall protocol for this study was approved by the Ethics Committee of the Faculty of Medicine and Health Sciences, Ghent University (Ref. No B67020084254), followed by a separate local ethical approval for each study site. For Brazil, approval was obtained from the Institutional Review Board from Centro de Pesquisas René Rachou (Ref. No 21/2008), for Cameroon from the National Ethics Committee (Ref. No 072/CNE/DNM08), for India from the Institutional Review Board of the Christian Medical College (Ref. No 6541), for Pemba Island (Tanzania; Ref. No 20) from the Zanzibar Health Research Council, and the Ministry of Health and Social Welfare, for Vietnam by the Ministry of Health of Vietnam. Parent(s)/guardians of participants in this study were asked to sign an informed consent document indicating that they understood both the purpose of and procedures required for the study, and that they were willing to let their child participate in the study. This study is registered under the ClinicalTrials.gov identifier NCT01087099.

### Study design

Details of the designs of both studies have been published elsewhere (study 1: [25]; study 2: [9,26]). Briefly, in both studies, at the baseline visit, school-aged children were asked to provide a fresh stool sample, after which they were treated with a single oral dose of 400 mg albendazole under supervision. The albendazole used in the different studies originated from the same production batch (GlaxoSmithKline Batch No 335726 (study 1) and L298 (study 2)) and was provided by WHO. At a follow-up visit, fourteen to 21 days after drug administration, a second stool sample was collected from all the children who had been found positive for any STH at baseline. All collected stool samples were processed to determine the FECs (expressed in EPG units) using microscopic methods and to estimate the DNA concentration of the different STH species (expressed as genome equivalents per ml of DNA extract (GE/ml)) applying qPCR. In study 1, both baseline and follow-up samples were analyzed by Kato-Katz (duplicate Kato-Katz), Mini-FLOTAC, FECPAK^G2^ and qPCR. In study 2, baseline samples were analyzed by single Kato-Katz and McMaster. Follow-up samples were processed using a single Kato-Katz only.

### Diagnostic methods

Detailed standard operating procedures (SOPs) for the different diagnostic methods have been published earlier [9,25]. Laboratory technicians were blinded to test results across the different diagnostic methods. In addition, ~10% of the stool samples analyzed by the microscopic methods were re-evaluated by a senior laboratory technician, and discrepancies (defined as suggested by Speich and co-workers [27]) were resolved. The details of the quality control performed during study 1 will be reported in a follow-up manuscript. Here we briefly mention the most important steps for each of the diagnostic methods.

For the single and duplicate Kato-Katz, slides were prepared and examined for the presence of STH eggs within 30–60 min following preparation. The results of slide A of the duplicate Kato-Katz (study 1) and the single Kato-Katz (study 2) represented the results of a single Kato-Katz, and egg counts were multiplied by 24 to obtain the FECs. The sum of the egg counts obtained after reading slide A and B (study 1) represented the results for duplicate Kato-Katz and were multiplied by 12 to obtain the FEC in EPG units. The duplicate Kato-Katz slides A and B were examined by two different laboratory technicians.

For Mini-FLOTAC, we homogenized two grams of fresh stool in 38 ml of flotation solution (saturated salt solution, density = 1.20) in the Fill-FLOTAC device [11]. After transferring the suspension into the two chambers of the Mini-FLOTAC, the device was placed on a horizontal surface for 10 min after which the reading disk was translated. Finally, STH eggs were quantified in both Mini-FLOTAC chambers, and multiplied by 10 to obtain FEC in EPG units. For FECPAK^G2^, we applied the SOP as described by Ayana et al. (2018) [7]. Briefly, 2 g of stools were homogenized in tap water in a Fill-FLOTAC device [11], after which the mixture was transferred into a FECPAK^G2^ sedimenter to allow STH eggs to sediment. The following day, the supernatant was poured off and saturated saline solution (specific density = 1.20) was added to the remaining slurry. The whole content of the sedimenter was then poured into a FECPAK^G2^ filtration unit from which two separate aliquots were taken and transferred into two wells of a FECPAK^G2^ cassette. Following an accumulation step of at least 20 minutes, the cassettes were placed in the Micro-I device for image capture. Subsequently, a technician identified and counted any STH eggs present in the images using specialized software. The number of eggs was multiplied by 34 to calculate the FEC in EPG units.

For qPCR, DNA was first extracted from an aliquot of 200 μl preserved stool suspension (0.5 ml in 1 ml 96%ethanol) [19]. These DNA samples were then analyzed for the presence and quantity of *Ascaris* spp, *Trichuris* spp, *Necator americanus* and *Ancylostoma* spp DNA (expressed as GE/ml) at the Laboratory for Medical Microbiology and Immunology (Elisabeth-Tweesteden Hospital, Tilburg, The Netherlands) using two multiplex qPCR assays ([19]; https://www.starworms.org/tools/overview/starworms-documents). The reported qPCR results for hookworms were calculated as the sum of GE/ml of the two hookworm species (*N*. *americanus* and *Ancylostoma* spp).

For the McMaster, two grams of stools were suspended in 30 ml of flotation solution (saturated salt solution, density = 1.20). The fecal suspension was poured three times through a wire mesh to remove large debris and then added to each of the two chambers of a McMaster slide. In each chamber, a volume of 0.5 ml of fecal suspension was examined for the presence of STH eggs. The number of eggs counted was multiplied by 50 to obtain the FEC in EPG units [9].

### Statistical analysis

Analyses were carried out on all *Ascaris*, *Trichuris* and hookworm cases. In the absence of a gold standard (method that has a sensitivity and specificity of 100%), we considered the composite reference standard (CRS) [28] as a proxy for a gold standard for identifying STH-positive cases. For study 1, this CRS method classified a sample as positive for an STH species if eggs or DNA were found by at least one of the diagnostic methods (FECPAK^G2^, duplicate Kato-Katz, Mini-FLOTAC and qPCR), and as negative if no eggs or DNA were found by any of the implemented methods. For this study, we considered test results of both baseline and follow-up samples of the different subjects. This approach allowed the artificial widening of the spectrum of possible FECs (high egg counts prior to drug administration and low egg counts following drug administration). For study 2, this CRS method classified a sample as infected with an STH species if eggs were found by at least one of the diagnostic methods (single Kato-Katz and McMaster), and as negative if no eggs were found by either method. For both datasets, a specificity of 100% was assumed for each method, as indicated by the morphology of the eggs or by the species/genus-specific qPCR assays [25;27].

Subsequently, we calculated receiver operator characteristic (ROC) curves to determine the method-specific threshold(s) that best distinguished M&HI infections from light intensity infections as defined by a single Kato-Katz and WHO thresholds (reference method) for each of the four diagnostic methods and the different STH species. For this the cutoff was determined by the point with maximal Youden’s index. For McMaster, Mini-FLOTAC and FECPAK^G2^ the thresholds were expressed in EPG units, whereas for qPCR they were expressed in GE/ml. The current WHO thresholds defining the intensity of infection when applying Kato-Katz across the different STH species are summarized in Table 1. Note that in the case of a false negative test result, we assumed light infection, and this for each of the different diagnostic methods.

In a next step, we verified whether applying the method-specific thresholds improved the agreement in classification of M&HI infections between the microscopic methods (Mini-FLOTAC, FECPAK^G2^ and McMaster) and the reference method (Kato-Katz and WHO thresholds). For this, we calculated 2 unweighted Fleiss-Cohen kappa statistics (κ) for each microscopic method and STH species separately: one for when the WHO thresholds were applied and one for when the method-specific thresholds were applied. The κ indicates a slight (0 ≤ κ <0.2), fair (0.2 ≤ κ <0.4), moderate (0.4 ≤ κ <0.6), substantial (0.6 ≤ κ <0.8) and an almost perfect agreement (κ ≥0.8). Any significant difference in both κ-statistics were verified by implementing a permutation test (5,000 iterations). We applied the Tukey method to account for three pair-wise comparisons (= three STH species per microscopic method), allowing to draw inferences on all STHs for each method.

In the absence of any WHO threshold for DNA-based methods, we assessed the agreement between Kato-Katz (based on the WHO thresholds) and qPCR (based on the method-specific thresholds) in classifying M&HI infections only. For this analysis, we calculated the κ (unweighted Fleiss-Cohen kappa statistic) for each STH species.

All statistical analyses were performed in R [29]. To calculate the ROC-curves and identify the optimal FEC or DNA concentration that maximized the correct classification both of low and M&H intensity infections, we used the ‘roc’ function within the ‘pROC’ [30]. The 95% CI around the probabilities of correct or false classifications were based on a bootstrap analysis using the ‘ci.thresholds’ function of the ‘pROC’ package.

## Results

### Study population

For study 1, complete data were available for 645 children across the three study sites (Ethiopia: 161 children; Lao PDR: 239 children; Tanzania (Pemba Island): 245 children). For study 2, complete data were available for 663 children across the five study sites (Brazil: 139; Cameroon: 89; India: 54; Tanzania (Pemba Island): 180 and Vietnam: 171). Based on the presence of STH-specific eggs or DNA, 863 stool samples tested positive for *Ascaris* (study 1: 540; study 2: 323), 1,243 samples for *Trichuris* (study 1: 889; study 2: 354) and 988 samples for hookworms (study 1: 675; study 2: 313) across the two studies. The qPCR results showed that all individuals from study 1 were infected with *N*. *americanus*, except for 8, where both *N*. *americanus* and *Ancylostoma* spp. were detected (all from one school in Pemba Island (Tanzania)).

S1 and S2 Figs describe the number of children recruited at baseline and follow-up, and the ultimate number of complete cases for the statistical data analysis for study 1 and 2, respectively. The description of the demographical parameters (e.g., age and sex) of the different cases is beyond the scope of the present study. For more details we refer the reader to [26], [9], [24] and [19]. The complete datasets of study 1 and Study 2 are made available in S1 and S2 Data, respectively.

### Infection intensity measured by four microscopic methods

Table 2 summarizes for each of the four microscopic methods the arithmetic mean of FECs and the proportion of false negative test results, light and M&H intensity infections (based on the WHO intensity thresholds). Compared to a single Kato-Katz (study 1: 10,000 EPG; study 2: 13,782 EPG), Mini-FLOTAC (6,404 EPG), FECPAK^G2^ (3,266 EPG) and McMaster (5,825 EPG) provided lower mean FECs for *Ascaris*. For *Trichuris*, both McMaster (592 *vs*. 766 EPG) and FECPAK^G2^ (286 *vs*. 1,917 EPG) provided lower FECs compared to Kato-Katz, whereas for the Mini-FLOTAC, the difference was negligible (1,838 *vs*. 1,917 EPG). For hookworms, all methods provided lower FECs than Kato-Katz (Mini-FLOTAC: 366 *vs*. 833 EPG; McMaster: 400 *vs*. 745 EPG; FECPAK^G2^: 275 *vs*. 833 EPG). These differences in mean FECs were also reflected in distinct differences in prevalence estimates for M&HI infections, microscopic methods resulting in lower prevalence estimates for M&HI infections when they were generating lower mean FECs. As an extreme example, the proportion of M&HI infections in study 1 based on a single Kato-Katz was 34.8% for *Ascaris*, 37.8% for *Trichuris* and 9.8% for hookworms, whereas for FECPAK^G2^ these numbers were 14.3%, 7.9% and 2.4%, respectively. Both Mini-FLOTAC and McMaster underestimated the proportion of M&HI infections for *Ascaris* (Mini-FLOTAC: 29.3% *vs*. 34.8%; McMaster: 30.0% *vs*. 41.2%) and hookworms (Mini-FLOTAC: 3.0% *vs*. 9.8%; McMaster: 3.5% *vs*. 7.7%), but presented a comparable proportion of M&HI *Trichuris* infections as obtained by Kato-Katz (Mini-FLOTAC: ~35%; McMaster: ~17%).

**Table 2:** The intensity of soil-transmitted helminth infections measured by four microscopic methods. The intensity of infections is summarized by arithmetic means of fecal egg counts (FECs; expressed as eggs per gram of stool (EPG)) and the proportion of light and moderate-to-high (M&H) intensity infections. For the classification of the infection intensity, we applied World Health Organization (WHO) infection intensity thresholds (see Table 1).

	Mean FEC (EPG)	False negative test results (%)	Infection of intensity based on WHO thresholds (%)	
			Light	M&H
**Study 1**				
*Ascaris* (n = 540)				
Single Kato-Katz	10,000	28.2	37.0	34.8
Mini-FLOTAC	6,404	36.6	34.1	29.3
FECPAK^G2^	3,266	41.2	44.6	14.3
*Trichuris* (n = 889)				
Single Kato-Katz	1,917	11.9	50.3	37.8
Mini-FLOTAC	1,838	8.5	56.4	35.1
FECPAK^G2^	286	40.1	52.0	7.9
Hookworms (n = 675)				
Single Kato-Katz	833	27.4	62.8	9.8
Mini-FLOTAC	366	26.0	71.0	3.0
FECPAK^G2^	275	47.5	50.1	2.4
**Study 2**				
*Ascaris* (n = 323)				
Single Kato-Katz	13,782	11.7	47.1	41.2
McMaster	5,825	25.4	44.6	30.0
*Trichuris* (n = 354)				
Single Kato-Katz	766	17.8	66.4	15.8
McMaster	592	19.5	63.6	16.9
Hookworms (n = 313)				
Single Kato-Katz	745	21.4	70.9	7.7
McMaster	400	28.0	68.4	3.5

### The degree of agreement in classifying M&HI infections between Kato-Katz and the other microscopic methods based on WHO thresholds

The probabilities for correctly classifying infection intensities based on WHO thresholds compared to Kato-Katz and the corresponding agreement with reference method are summarized for Mini-FLOTAC, FECPAK^G2^ and McMaster in Table 3. Based on the observed differences in mean FECs, it is not surprising that the three microscopic methods mainly failed to correctly classify M&HI infections, and that only in a minority of the cases were low intensity infections falsely classified as M&H. More than 14% of the M&HI infections were falsely classified as low, whereas fewer than 10% of the low intensity infections were misclassified. The proportion of correctly classified infection intensities was generally higher for *Ascaris* (63.2%– 80.3%) and Mini-FLOTAC (27.3%– 85.1%). The lowest values were observed for hookworms (21.2%– 33.3%) and FECPAK^G2^ (19.6%– 39.4%). These observations were also reflected in the κ-statistic. For *Ascaris* infections, there was a moderate to almost perfect agreement, whereas for hookworms the agreement was moderate at best. For Mini-FLOTAC, the agreement was almost perfect for *Ascaris* and *Trichuris*, but fair for hookworms. For FECPAK^G2^, the agreement was moderate for *Ascaris*, but fair for the other STHs. For McMaster the agreement was moderate across all STHs.

**Table 3:** The probabilities to correctly classifying infections intensities based on WHO thresholds. The World Health Organization (WHO) thresholds for classifying the intensity of infections into light, moderate and heavy are summarized in Table 1. The Kappa statistic reflects represents the degree of agreement in classifying M&HI infections between the microscopic methods (Mini-FLOTAC, FECPAK^G2^ and McMaster) and the reference method (Kato-Katz and WHO thresholds). 95% CI: 95% confidence interval; M&H: moderate-to-heavy.

	Proportion (%) of M&HI infections correctly classified as M&H (95% CI)	Proportion (%) of light intensity infections falsely classified as M&H (95% CI)	Kappa statistic (95% CI)tand and the interpretation	
***Ascaris***				
Mini-FLOTAC	80.3 (74.6; 86.0)	2.0 (0.5; 3.5)	0.81 (0.76; 0.87)	Almost perfect
FECPAK^G2^	39.4 (32.4; 46.4)	0.9 (0; 1.9)	0.45 (0.37; 0.52)	Moderate
McMaster	63.2 (55.0; 71.4)	6.8 (3.2; 10.4)	0.59 (0.50; 0.68)	Moderate
***Trichuris***				
Mini-FLOTAC	85.1 (81.3; 88.9)	4.7 (2.9; 6.5)	0.81 (0.78; 0.86)	Almost perfect
FECPAK^G2^	19.6 (15.4; 23.8)	0.7 (0; 1.4)	0.22 (0.17; 0.27)	Fair
McMaster	57.1 (44.1; 70.1)	9.4 (6.1; 12.7)	0.46 (0.34; 0.59)	Moderate
**Hookworm**				
Mini-FLOTAC	27.3 (16.6; 38.0)	0.3 (0; 0.7)	0.39 (0.26; 0.52)	Fair
FECPAK^G2^	21.2 (11.3; 31.1)	0.3 (0; 0.7)	0.32 (0.19; 0.44)	Fair
McMaster	33.3 (14.4; 52.2)	1.0 (0; 2.1)	0.43 (0.22; 0.64)	Moderate

### The degree of agreement in classifying M&HI infections between Kato-Katz and the other diagnostic methods based on method-specific thresholds

The different ROC-curves are presented in S3 Fig. Table 4 summarizes the method-specific thresholds that maximize the percentage of correctly classified M&HI infections, while minimizing the percentage of falsely classified light intensity infections. With the exception of the McMaster for the quantification of *Ascaris* infections, for which the ROC-analysis provided two potential thresholds, only one threshold was identified for each STH species and diagnostic method. Overall, there was considerable variation in the proportion of correctly classified M&HI infections and the proportion of falsely classified light intensity infections across STH species and diagnostic methods. The proportion of M&HI infections was highest for *Ascaris*. For this STH species, the proportion of samples with correctly classified infection intensities did not drop below 80%, ranging from 83.5% to 94.7%, whereas for *Trichuris* the proportion ranged from 78.6% to 93.5%, and for hookworms it did not even reach 90% (78.8%– 87.9%). The proportion of correctly classified M&HI infections was highest for Mini-FLOTAC across all STH species. For this diagnostic method, the proportion of correctly classified M&HI infections did not drop below 85%. The lowest values for *Ascaris*, *Trichuris* and hookworm were observed when applying qPCR (83.5%), FECPAK^G2^ (78.6%) and qPCR (78.8%), respectively. The proportion of falsely classified light intensity infections was highest for *Trichuris*. For this STH species, the proportion exceeded 20% in three out of the four diagnostic methods (FECPAK^G2^, McMaster and qPCR), ranging from 9.4% to 25.0%, whereas for both *Ascaris* and hookworms it exceeded 20% for one diagnostic method only (McMaster), ranging from 8.0% to 21.2% and from 9.2 to 20.8%, respectively. Across diagnostic methods, the proportion of falsely classified light intensity infections as M&H was lowest for Mini-FLOTAC across all STH species (8–9.4%), and highest for McMaster (*Ascaris*: 21.1%; hookworm: 20.8%) and qPCR (*Trichuris*: 25.0%). These observations were also reflected in the κ-statistic. For Mini-FLOTAC, there was an almost perfect agreement for all STHs, except for hookworms. For this STH, the agreement was moderate. For the other diagnostic methods, there was a substantial agreement for *Ascaris* infections and a moderate agreement for *Trichuris*. For hookworms, there was a moderate agreement for FECPAK^G2^ and qPCR, but a fair agreement for McMaster.

**Table 4:** Method-specific thresholds and the corresponding probabilities of classifying moderate-to-heavy and light intensity infections. The method-specific thresholds were derived from receiver operating (ROC) curves for Mini-FLOTAC, FECPAK^G2^, McMaster and qPCR, separately. In these ROC analyses, the reference classification of moderate-to-heavy intensity (M&HI) infections was based on the Kato-Katz and the World Health Organization infection intensity thresholds (Table 1). They maximize the percentage of correctly classified M&HI infections while minimizing the percentage of light intensity infections falsely classified as M&H. The thresholds are expressed as eggs per gram of stool (EPG) for Mini-FLOTAC, FECPAK^G2^ and McMaster, and as the number of genome equivalents per ml of DNA (GE/ml) for qPCR. The Kappa reflects the degree of agreement in classifying M&HI infections based on the method-specific thresholds between the microscopic methods (Mini-FLOTAC, FECPAK^G2^ and McMaster) and the reference method (Kato-Katz and WHO thresholds). 95% CI: 95% confidence interval; M&H: moderate-to-heavy.

	Threshold (EPG or GE/ml)	Proportion (%) of M&HI infections correctly classified (95% CI)	Proportion (%) of light intensity infections falsely classified as M&H (95% CI)	Kappa (95% CI)	
***Ascaris***					
Mini-FLOTAC	≥2,980	94.7 (91.5; 97.9)	8.0 (5.1; 10.8)	0.85 (0.80; 0.89)	Almost perfect
FECPAK^G2^	≥289	91.0 (86.7; 94.7)	15.9 (12.2; 19.6)	0.72 (0.66; 0.78)	Substantial
McMaster	≥1,775	90.2 (85.0; 94.7)	21.1 (15.8; 26.8)	0.67 (0.59; 0.75)	Substantial
	≥2,500	85.0 (78.2; 90.2)	15.8 (11.1; 21.1)	0.68 (0.60; 0.76)	Substantial
qPCR	≥7,332	83.5 (78.2; 88.3)	12.2 (8.8; 15.6)	0.70 (0.64; 0.77)	Substantial
***Trichuris***					
Mini-FLOTAC	≥715	93.5 (90.8; 95.8)	9.4 (7.1; 11.9)	0.83 (0.79; 0.86)	Almost perfect
FECPAK^G2^	≥85	78.6 (74.1; 82.7)	20.4 (17.0; 23.7)	0.57 (0.51; 0.62)	Moderate
McMaster	≥425	82.1 (71.4; 91.1)	24.2 (19.1; 29.2)	0.40 (0.30; 0.50)	Moderate
qPCR	≥872	87.2 (83.3; 90.8)	25.0 (21.5; 28.8)	0.59 (0.54; 0.64)	Moderate
**Hookworms**					
Mini-FLOTAC	≥510	87.9 (78.8; 95.5)	9.2 (7.1; 11.5)	0.59 (0.51; 0.68)	Moderate
FECPAK^G2^	≥221	87.9 (78.8; 95.5)	13.6 (11.0; 16.3)	0.49 (0.41; 0.58)	Moderate
McMaster	≥375	79.2 (62.5; 91.7)	20.8 (16.3; 25.6)	0.28 (0.17; 0.40)	Fair
qPCR	≥111,518	78.8 (68.2; 87.9)	11.5 (8.9; 14.0)	0.49 (0.40; 0.58)	Moderate

### Comparison between WHO and method-specific thresholds for the classification of M&HI infections based on microscopic methods

**Fig 1:**
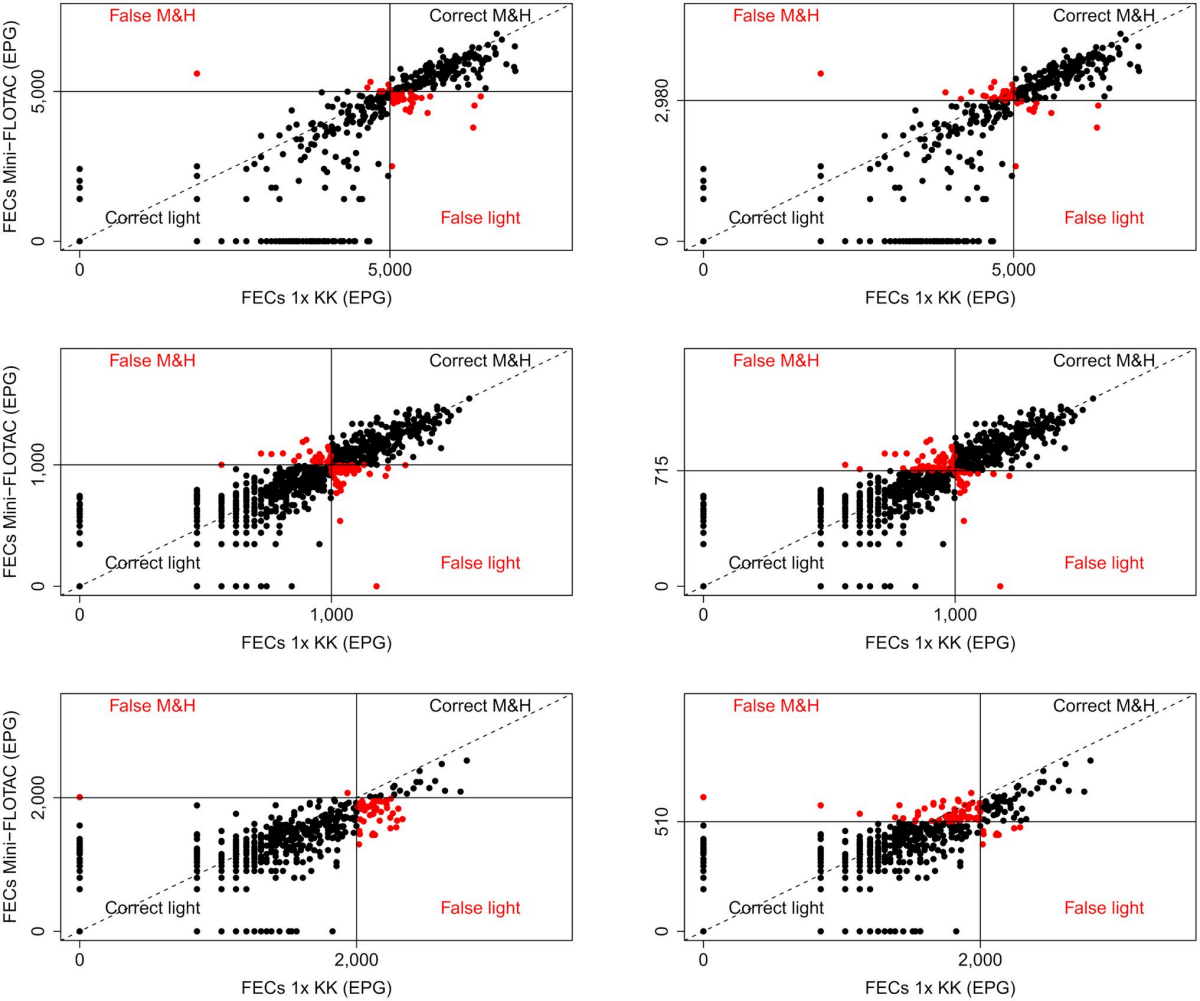
Agreement in classifying infection intensities for Mini-FLOTAC when applying WHO and method-specific thresholds. The scatter plots represent the degree of agreement between Mini-FLOTAC and a single Kato-Katz (1x KK; reference method) in classifying infection intensity based on World Health Organization (WHO; Table 1) or the method-specific thresholds (Table 4) for *Ascaris*, *Trichuris* and hookworms. The full black dots indicate that intensity of infection was correctly classified as light (bottom left quadrant) and moderate-to-heavy (M&H; top right quadrant). The red dots indicate that intensity of infection was falsely classified as light (bottom right quadrant) and M&H (top left quadrant). The full lines represent the thresholds, the dashed lined represents the line of equality.

**Fig 2:**
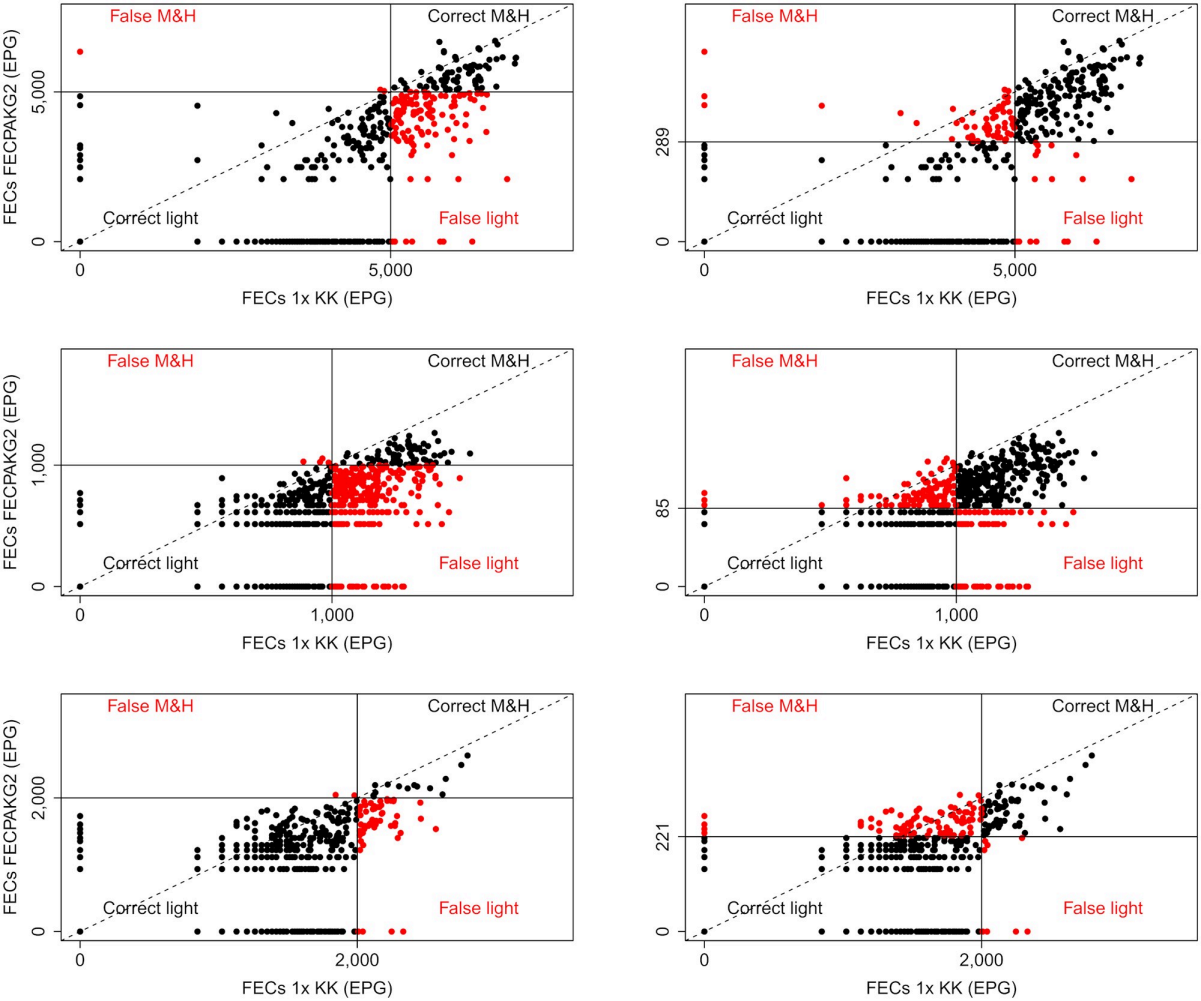
Agreement in classifying infection intensities for FECPAK^G2^ when applying WHO and method-specific thresholds. The scatter plots represent the degree of agreement between FECPAK^G2^ and a single Kato-Katz (1x KK; reference method) in classifying infection intensity based on World Health Organization (WHO; Table 1) or the method-specific thresholds (Table 4) for *Ascaris*, *Trichuris* and hookworms. The full black dots indicate that intensity of infection was correctly classified as light (bottom left quadrant) and moderate-to-heavy (M&H; top right quadrant). The red dots indicate that intensity of infection was falsely classified as light (bottom right quadrant) and M&H (top left quadrant). The full lines represent the thresholds, the dashed line represents the line of equality.

**Fig 3:**
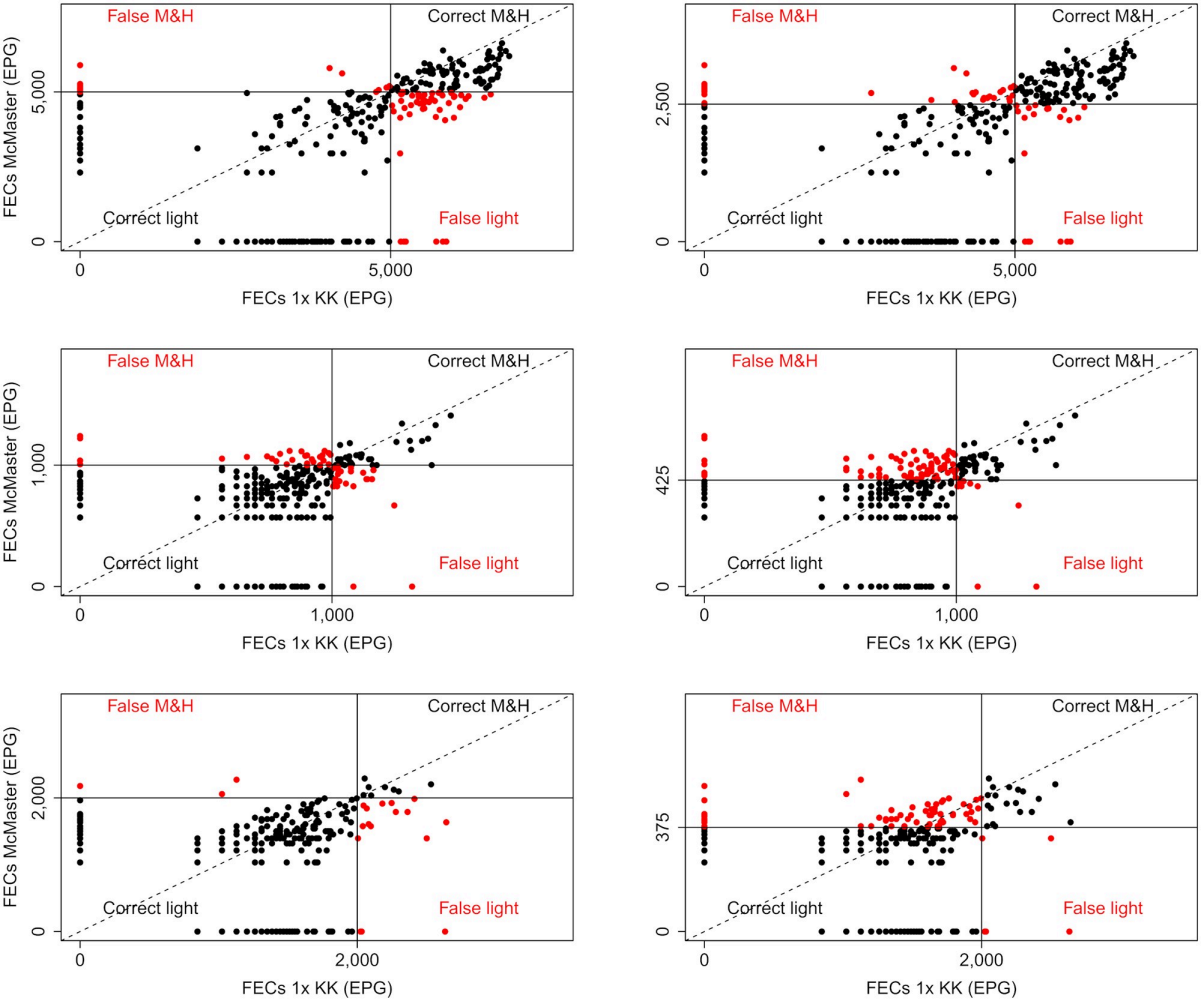
Agreement in classifying infection intensities for McMaster when applying WHO and method-specific thresholds. The scatter plots represent the degree of agreement between McMaster and a single Kato-Katz (1x KK; reference method) in classifying infection intensity based on either World Health Organization (WHO; Table 1) and method-specific thresholds (Table 4) for *Ascaris*, *Trichuris* and hookworms. The full black dots indicate that intensity of infection was correctly classified as light (bottom left quadrant) and moderate-to-heavy (M&H; top right quadrant). The red dots indicate that intensity of infection was falsely classified as light (bottom right quadrant) and M&H (top left quadrant). The full lines represent the thresholds, the dashed line represents the line of equality.

**Fig 4:**
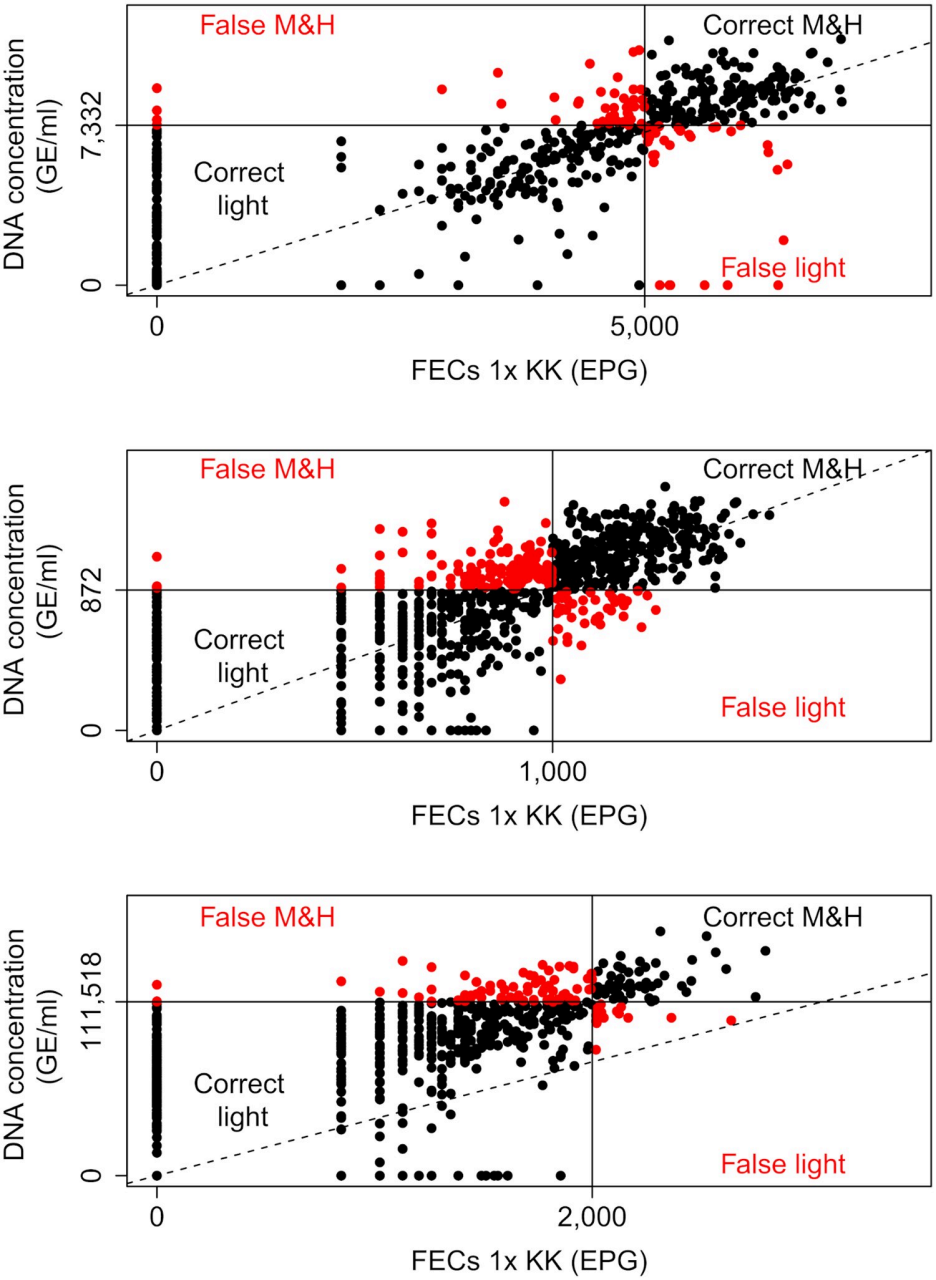
Agreement in classifying infection intensities between Kato-Katz and qPCR. The scatter plots represent the de degree agreement between qPCR and a single Kato-Katz (1x KK; reference method) in classifying infection intensity cases based World Health Organization (WHO; Table 1) and method-specific thresholds (Table 4) for *Ascaris*, *Trichuris* and hookworms. The full black dots indicate that intensity of infection was correctly classified as light (bottom left quadrant) and moderate-to-heavy (M&H; top right quadrant). The red dots indicate that intensity of infection was falsely classified as light (bottom right quadrant) and M&H (top left quadrant). The full lines represent the thresholds, the dashed line represents the line of equality.

As illustrated by Figs 1–3 and by Tables 3 and 4, a higher percentage of M&HI infections were correctly classified when applying the method-specific thresholds for Mini-FLOTAC, FECPAK^G2^ and McMaster, respectively. Yet, there was also an increased number of incorrectly classified low intensity infections. Overall, the method-specific thresholds significantly improved the agreement with the reference method, but differences across both diagnostic methods and STHs were observed. For FECPAK^G2^, a significantly improved agreement was observed across all STHs (*Ascaris*: κ = 0.45 [moderate] → κ = 0.72 [substantial], *p* <0.01; *Trichuris*: κ = 0.22 [fair] → κ = 0.57 [moderate], *p* <0.001; hookworms: κ = 0.32 [fair] → κ = 0.49 [moderate], *p* = 0.04). For Mini-FLOTAC, a significantly improved agreement was only observed for hookworms (κ = 0.39 [fair] → κ = 0.59 [moderate], *p* = 0.01), for the other STHs the agreement remained unchanged (*Ascaris*: κ = 0.81 [almost perfect] *vs*. κ = 0.85 [almost perfect], *p* = 0.75; *Trichuris*: κ = 0.81 [almost perfect] *vs*. κ = 0.80 [almost perfect], *p* = 0.98). For McMaster, the method-specific thresholds did not significantly improve the detection of M&HI infections for any of the STHs. The κ-statistic changed from 0.59 (moderate) to 0.70 (substantial) for *Ascaris* (*p* = 0.40), and remained roughly unchanged for *Trichuris* (κ = 0.46 [moderate] *vs*. κ = 0.59 [moderate], *p* = 0.71). For hookworms, the κ-statistic even dropped from 0.43 (moderate) to 0.28 (fair), though not significantly (*p* = 0.16). The degree of agreement in classifying infection intensities for qPCR when applying WHO and method-specific thresholds is illustrated in Fig 4.

## Discussion

The prevalence of M&HI infections is a key indicator for measuring the success of large-scale deworming programs for STHs [3]. Although newly introduced microscopy-(FECPAK^G2^, McMaster and Mini-FLOTAC) and DNA-based (qPCR) methods have important advantages, it remains unclear whether applying current WHO thresholds to these methods allows for a reliable classification of M&HI infections. In the present study, we applied the current WHO thresholds for classifying FECs into light or M&H intensity infections for each of the three microscopic methods. We then verified whether method-specific thresholds determined in this study improved the classification of M&HI infections compared to when WHO thresholds were applied. For qPCR, we determined the corresponding qPCR-specific thresholds that maximize the degree of agreement in classifying infection intensity with values derived by the Kato-Katz method. To our knowledge, this is the first study to explore method-specific thresholds for Mini-FLOTAC, FECPAK^G2^, McMaster and qPCR in a large study population (1,300 infected subjects) from seven STH endemic countries where large-scale deworming programs are ongoing (Brazil, Cameroon, Ethiopia, India, Lao PDR, Tanzania and Vietnam).

### Elimination of STH as a public health problem may be falsely declared when applying the WHO thresholds to FECs obtained through microscopic methods other than Kato-Katz

Overall, our results demonstrate that applying WHO thresholds on FECs obtained using Mini-FLOTAC, FECPAK^G2^ and McMaster mainly misclassified M&HI as low intensity, and to a lesser extent low intensity infection as M&H, which in turn resulted in an underestimation of the prevalence of M&HI infections (see Table 2). Our data showed also that the probability of correctly classifying M&HI when compared to Kato-Katz varied across the different diagnostic methods and STH species (Table 2), the probability of misclassification being most pronounced for FECPAK^G2^ and hookworms. These variations across diagnostic methods and STHs are the result of variation in the method-specific FECs (Table 1). Although it is beyond the scope of the present paper to explain these variations in FECs (for a more detailed discussion on this topic we refer to the work of Cools and colleagues [19]), our findings emphasize that applying WHO thresholds on microscopic methods other than Kato-Katz is highly likely to lead to incorrect conclusions about the elimination of soil-transmitted helminthiasis as a public health problem. It is therefore of utmost importance that M&HI infection prevalence estimates based on methods other than Kato-Katz are interpreted with caution.

### Towards method-specific thresholds for all STH species

Although our results strongly indicate that method-specific thresholds should be applied rather than the current WHO thresholds, some relevant aspects need to be carefully considered. First it is important to state that these method-specific thresholds have not yet been fully validated. In the present study, we merely made initial estimates for these thresholds across different data sets. A thorough validation would require an examination of the suitability of these method-specific thresholds across different datasets where Kato-Katz is used as comparator (e.g., data sets reviewed by Nikolay et al., 2014 [8]; [17]). For this, it will be important to also consider the probability of correctly classifying light and M&HI infections. This is because, the method-specific thresholds do not allow for a perfect classification of the infection intensities (see Table 4), and hence uncorrected prevalence estimates of M&HI infections will be biased (see S1 Table). This phenomenon of biased estimates in prevalence of M&HI infections is very much comparable to estimating true prevalence with an imperfect diagnostic method test. Formulae exist for estimating both the true prevalence and corresponding 95% CI when sensitivity (probability of correctly classifying M&HI infections) and specificity (probability of correctly classifying low intensity infections) of the test are known [31]. In S1 Table we applied these formulae, indicating that the uncorrected prevalence estimates are indeed biased (particularly for hookworms), but as expected, are virtually identical to those of a single Kato-Katz when we account for imperfect thresholds. Second, the number of M&HI hookworms infections was low (<100 cases) across all diagnostic methods (study 1: 66 out of 675; study 2: 24 out of 313). For *Trichuris*, the numbers were also low for McMaster (study 2: 56 out of 354), and hence more data are required to calculate and recommend robust method-specific thresholds for hookworm infections and the McMaster method. Third, FECPAK^G2^, McMaster and Mini-FLOTAC concentrate eggs by using suspended stools in a solution with a higher density than that of the STH eggs (hence eggs should float to the surface of the chambers/wells). In the present study, saturated salt solution was used across all methods, but it has been shown that other flotation solutions (e.g zinc sulphate) with a different density will result in different FECs [15,32]. As a consequence of these differences in FECs across flotation solutions, the method-specific thresholds derived in the current study can only be applied when saturated salt solution is used. Finally, in the present study *Necator* was the most prevalent hookworm species. However, there is a distinct difference in fecundity (resulting in a difference in DNA concentration) between different hookworm species [33], and hence the current qPCR thresholds may not be applicable for *Ancylostoma* spp.

### Need for a consensus on how to report qPCR data

Although there have been a number of attempts to determine qPCR-specific thresholds for classifying the intensity of infections, these were not based on clinical samples (seeding experiments), not linked to Kato-Katz results [22] or were based on Ct-values [34], and hence it is difficult to compare the proposed thresholds with those identified in the present study. Overall, the agreement in classifying light and M&H intensity infections was moderate for hookworms and *Trichuris*, and extremely good for *Ascaris*. A key obstacle that impedes further validation of these thresholds, and ultimately recommending them, is the absence of a universal unit to express qPCR data. In the present study, we expressed the outcome of qPCR as GE/ml. In contrast to other units, this allows for both an absolute quantification of DNA present in the sample (*vs*. Ct-values), is not affected by both the copy number of the target gene (e.g., single copy *vs*. multi-copy target genes) and allows for comparisons between laboratories (which is not possible when using Ct-values). For a more detailed review on the need for a universal unit and the argumentation for GE/ml as a universal unit, we refer the reader to [35] and Cools et al. (under review), respectively.

### Diagnostic methods need to be evaluated for the assessment of M&HI infections

In a broader context, the present study also underscores the need to align the evaluation of diagnostic methods with current STH program goals. Too often, newly introduced methods are evaluated based on their clinical sensitivity only, while this parameter is probably less important for the assessment of M&HI infections. For example, FECPAK^G2^ has a high clinical sensitivity (>90%) for the detection of M&HI infections for all three STHs [19] but fails to accurately classify infection intensities when applying the WHO thresholds. Moreover, it will be important to define objectively minimal and ideal (optimistic) criteria that a diagnostic method should meet (referred to as *target product profiles* (TPPs)). Although a group of key STH diagnostic experts recently published a framework in which they defined TPPs across different phases of a STH control program, criteria for classifying intensity of infection were missing [36].

In conclusion, our results indicate that method-specific thresholds improved the classification of M&HI infections, but we stress that validation studies are required before they can be recommended for general use in assessing M&HI infections in programmatic settings. The study also highlights the need (i) to agree on an absolute universal unit for qPCR, (ii) to align the evaluation of diagnostic methods with current STH program goals, and (iii) to define minimal and ideal (optimistic) criteria that diagnostic methods should meet in order to assess M&HI infections reliably.

## Supporting information

S1 Fig Number of subjects in study 1 withheld at recruitment, baseline and follow-up, and for the statistical data analysis. STH: soil-transmitted helminth; n: number of subjects; FECs: fecal egg counts expressed in eggs per gram of stool (EPG), 2x KK: duplicate Kato-Katz.
(TIFF)

S2 Fig Number of subjects in study 2 withheld at recruitment, baseline and follow-up, and for the statistical data analysis. (PDF)

S3 Fig Receiver operating curves to classify moderate-to-heavy soil-transmitted helminth intensity infections applying four different diagnostic methods. The panels present the receiver operating curves (ROC) for classifying moderate-to-heavy intensity (M&HI) *Ascaris*, *Trichuris* and hookworm infections for Mini-FLOTAC (Panel A), FECPAK^G2^ (Panel B), McMaster (Panel C) and qPCR (Panel D). ‘•’ or ‘°’ represent the fecal egg count (expressed as eggs per gram of stool) or DNA concentration (genome equivalents per ml of DNA extract) that maximizes the percentage of M&HI infections correctly classified while minimizing the percentage of falsely classifying light intensity infections as moderate-to-heavy (M&H). AUC: area under the curve; 95% CI: 95% confidence intervals based on bootstrap analysis.
(PDF)

S1 Table The uncorrected and corrected prevalence of moderate-to-heavy intensity infections based on method-specific thresholds. The table reports both the uncorrected and corrected prevalence of moderate-to-heavy (M&H) intensity infections for Mini-FLOTAC, FECPAK^G2^, McMaster and qPCR. The uncorrected prevalence equals to the number of samples classified as M&H intensity based on the method-specific thresholds (Table 4). The corrected prevalence and the corresponding 95% confidence intervals (95% CI) are based on the formulae described by Diggle (2011) [33].
(DOCX)

S1 Data Complete dataset study 1. (XLSX)

S2 Data Complete dataset study 2. (XLS)
