# Supplementary figures and images for: Identifying thresholds for classifying moderate-to-heavy soil-transmitted helminth intensity infections for FECPAKG2, McMaster, Mini-FLOTAC and qPCR
Source: PLoS Negl Trop Dis. 2020 Jul 2;14(7):e0008296. doi: 10.1371/journal.pntd.0008296 (PMC7413557; doi:10.1371/journal.pntd.0008296)

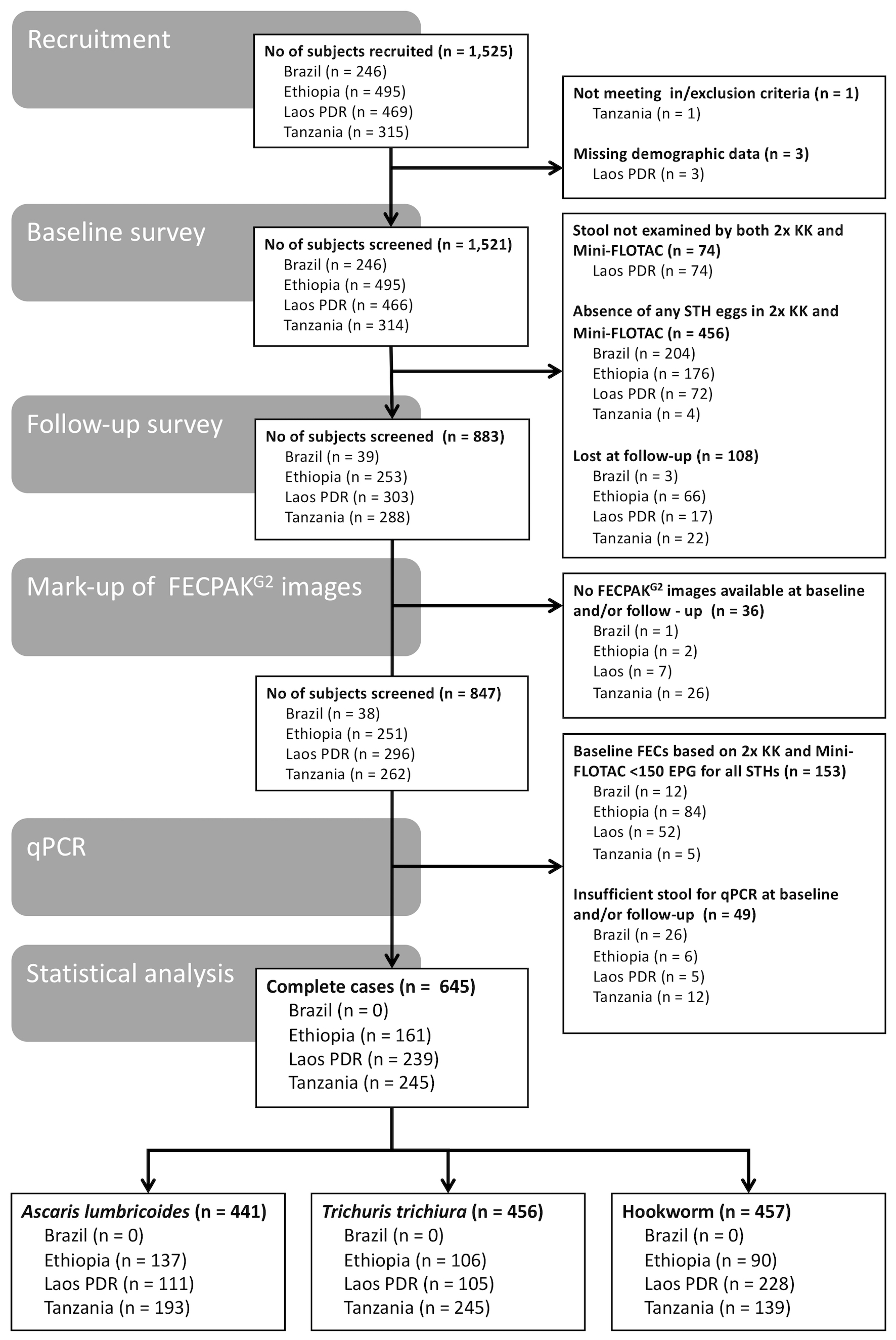

Supplement: S1 Fig — STH: soil-transmitted helminth; n: number of subjects; FECs: fecal egg counts expressed in eggs per gram of stool (EPG), 2x KK: duplicate Kato-Katz. (TIFF) [file pntd.0008296.s001.tiff]

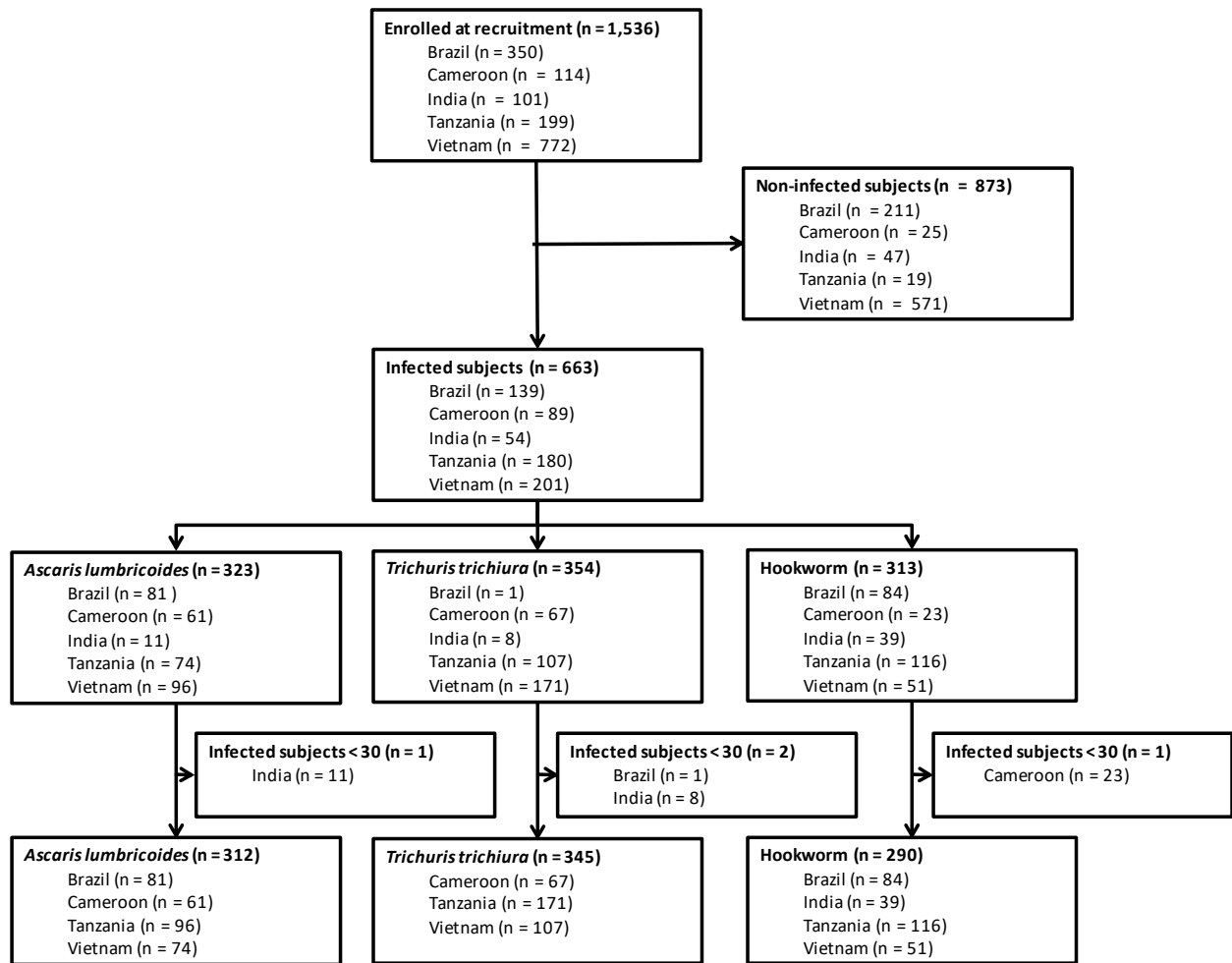

Supplement: S2 Fig — (PDF) [file pntd.0008296.s002.pdf]
