# Supplementary material for: Identifying thresholds for classifying moderate-to-heavy soil-transmitted helminth intensity infections for FECPAKG2, McMaster, Mini-FLOTAC and qPCR
Source: PLoS Negl Trop Dis. 2020 Jul 2;14(7):e0008296. doi: 10.1371/journal.pntd.0008296 (PMC7413557; doi:10.1371/journal.pntd.0008296)
Supplement: S3 Fig — The panels present the receiver operating curves (ROC) for classifying moderate-to-heavy intensity (M&HI) Ascaris, Trichuris and hookworm infections for Mini-FLOTAC (Panel A), FECPAKG2 (Panel B), McMaster (Panel C) and qPCR (Panel D). ‘•’ or ‘°’ represent the fecal egg count (expressed as eggs per gram of stool) or DNA concentration (genome equivalents per ml of DNA extract) that maximizes the percentage of M&HI infections correctly classified while minimizing the percentage of falsely classifying light intensity infections as moderate-to-heavy (M&H). AUC: area under the curve; 95% CI: 95% confidence intervals based on bootstrap analysis. (PDF) [file pntd.0008296.s003.pdf]

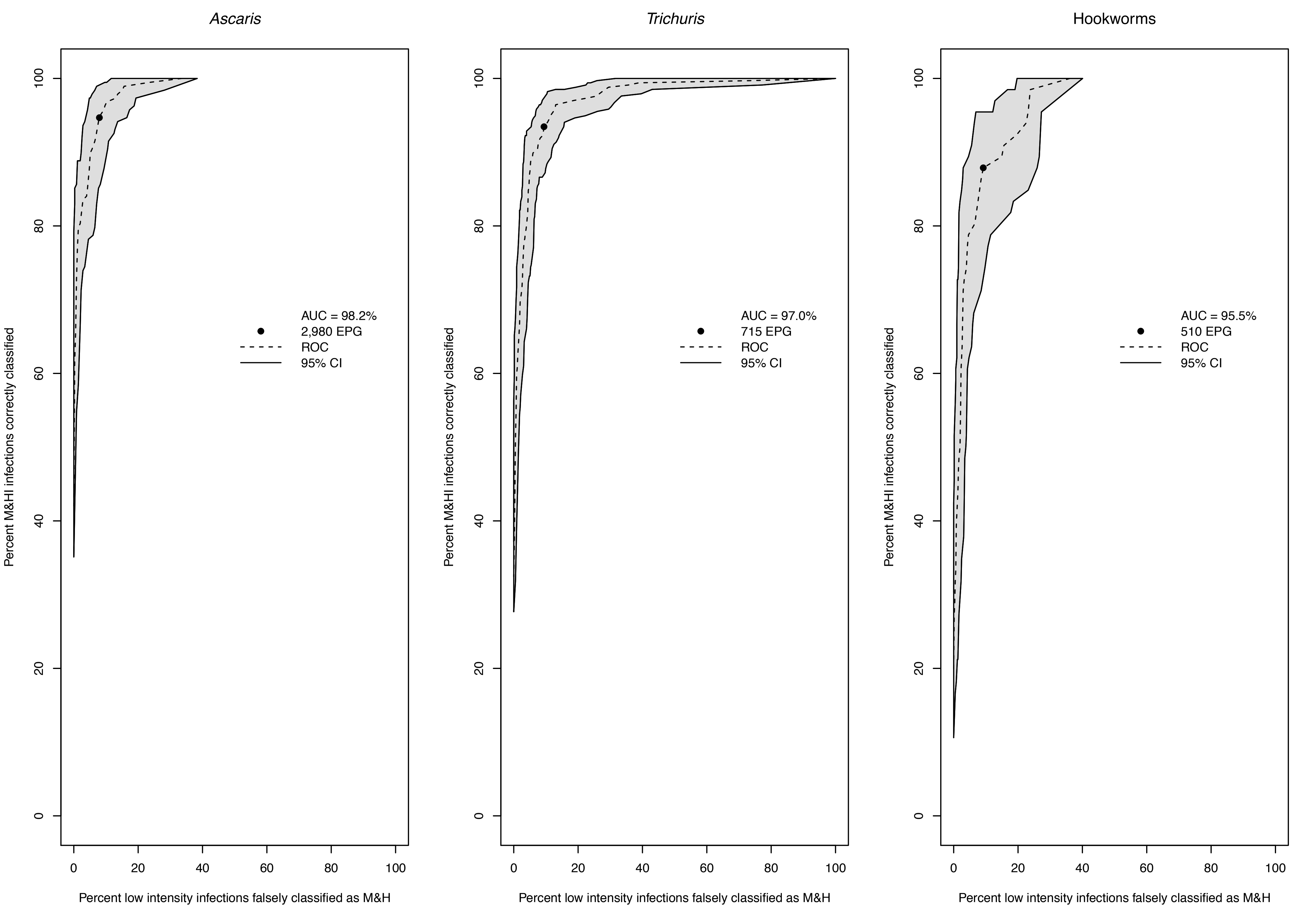

Ascaris
Trichuris
Hookworms
Percent M&HI infections correctly classified
Percent low intensity infections falsely classified as M&H
AUC = 98.2%
2,980 EPG
ROC
95% CI
AUC = 97.0%
715 EPG
AUC = 95.5%
510 EPG
0
20
40
60
80
100

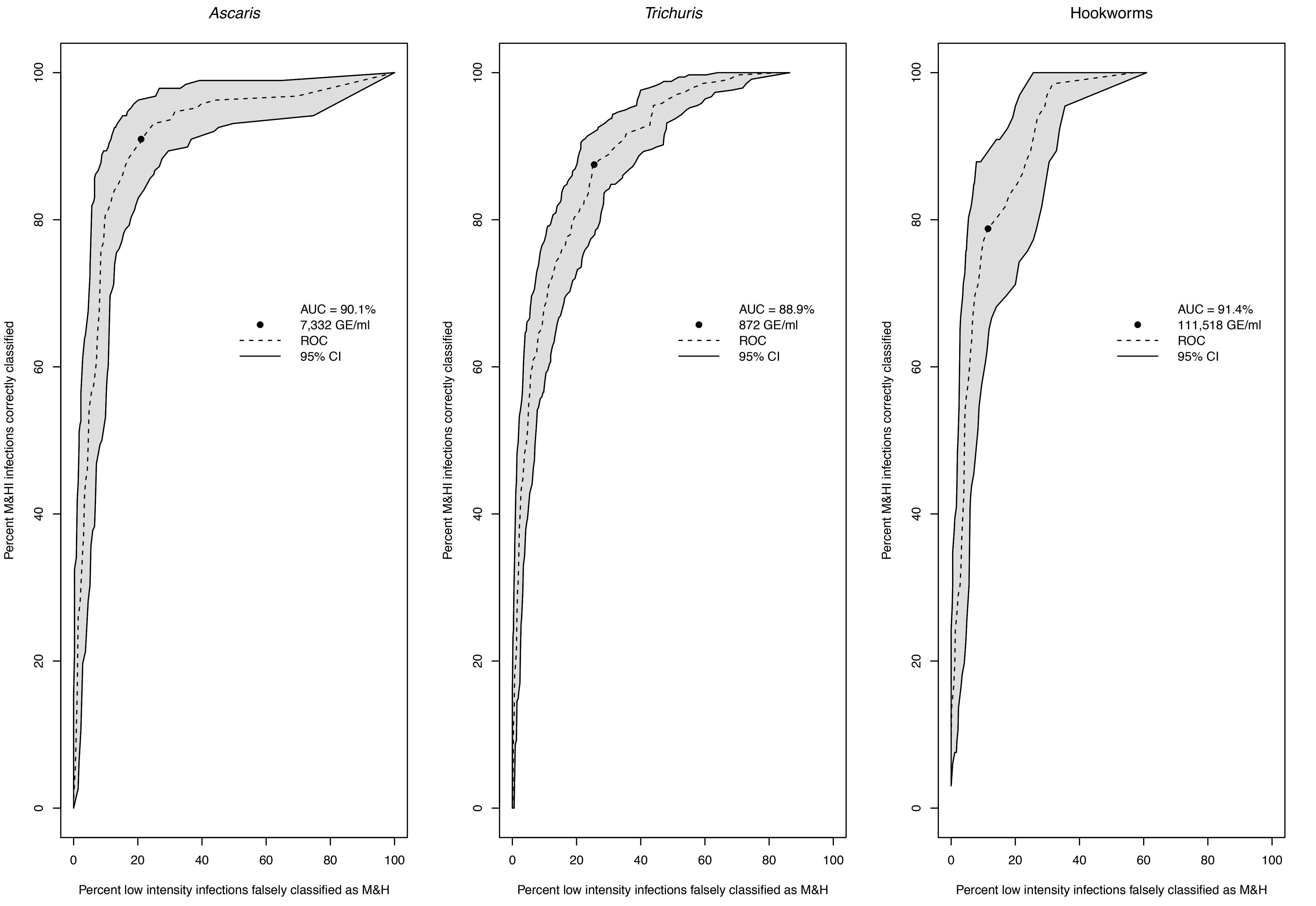
Ascaris
AUC = 90.1%
7,332 GE/ml
ROC
95% CI
Trichuris
AUC = 88.9%
872 GE/ml
ROC
95% CI
Hookworms
AUC = 91.4%
111,518 GE/ml
ROC
95% CI
Percent M&HI infections correctly classified
Percent low intensity infections falsely classified as M&H

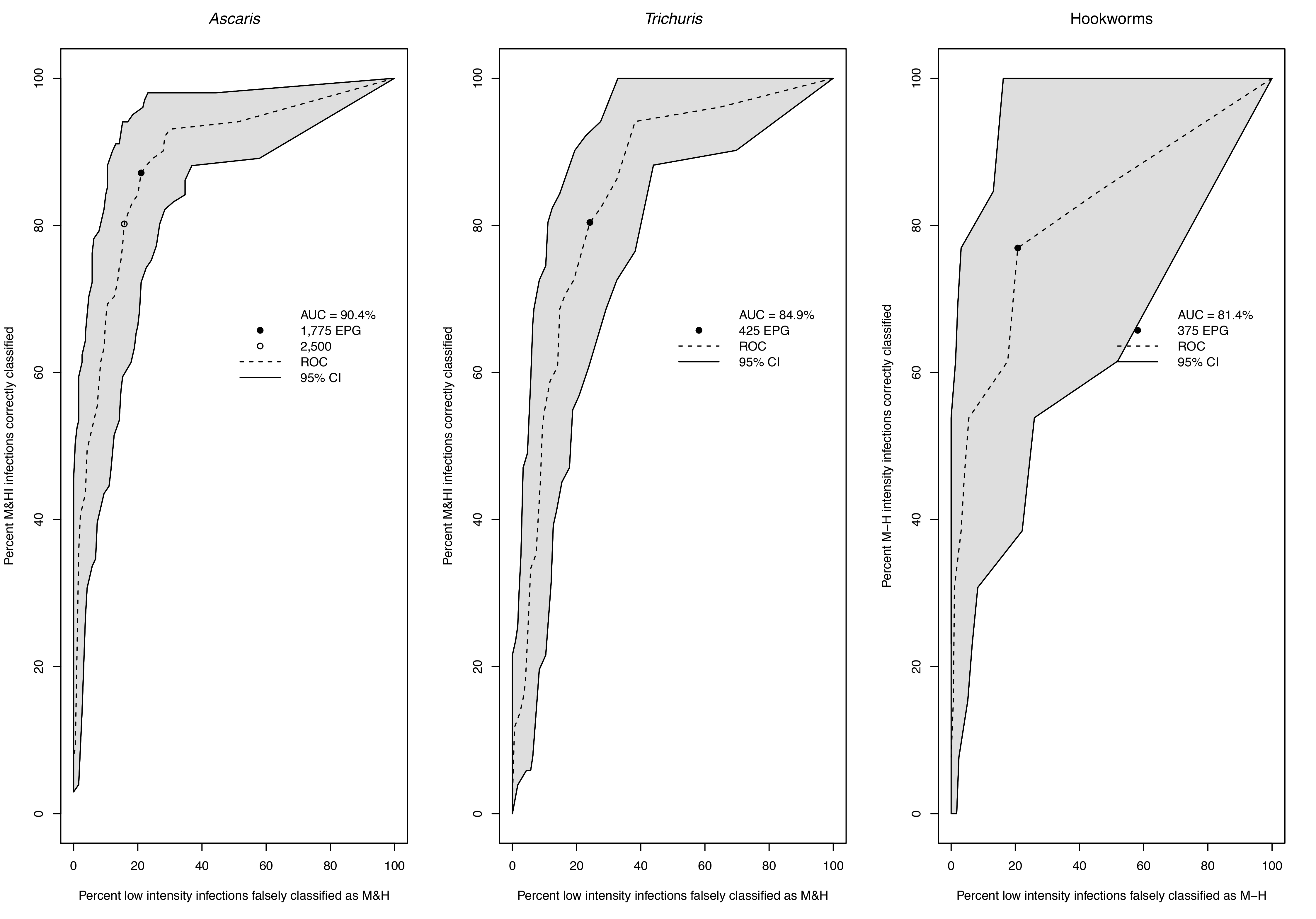
Ascaris
Trichuris
Hookworms
AUC = 90.4%
1,775 EPG
2,500
ROC
95% CI
AUC = 84.9%
425 EPG
ROC
95% CI
AUC = 81.4%
375 EPG
ROC
95% CI
Percent M&HI infections correctly classified
Percent M&HI infections correctly classified
Percent M–H intensity infections correctly classified
Percent low intensity infections falsely classified as M&H
Percent low intensity infections falsely classified as M&H
Percent low intensity infections falsely classified as M–H

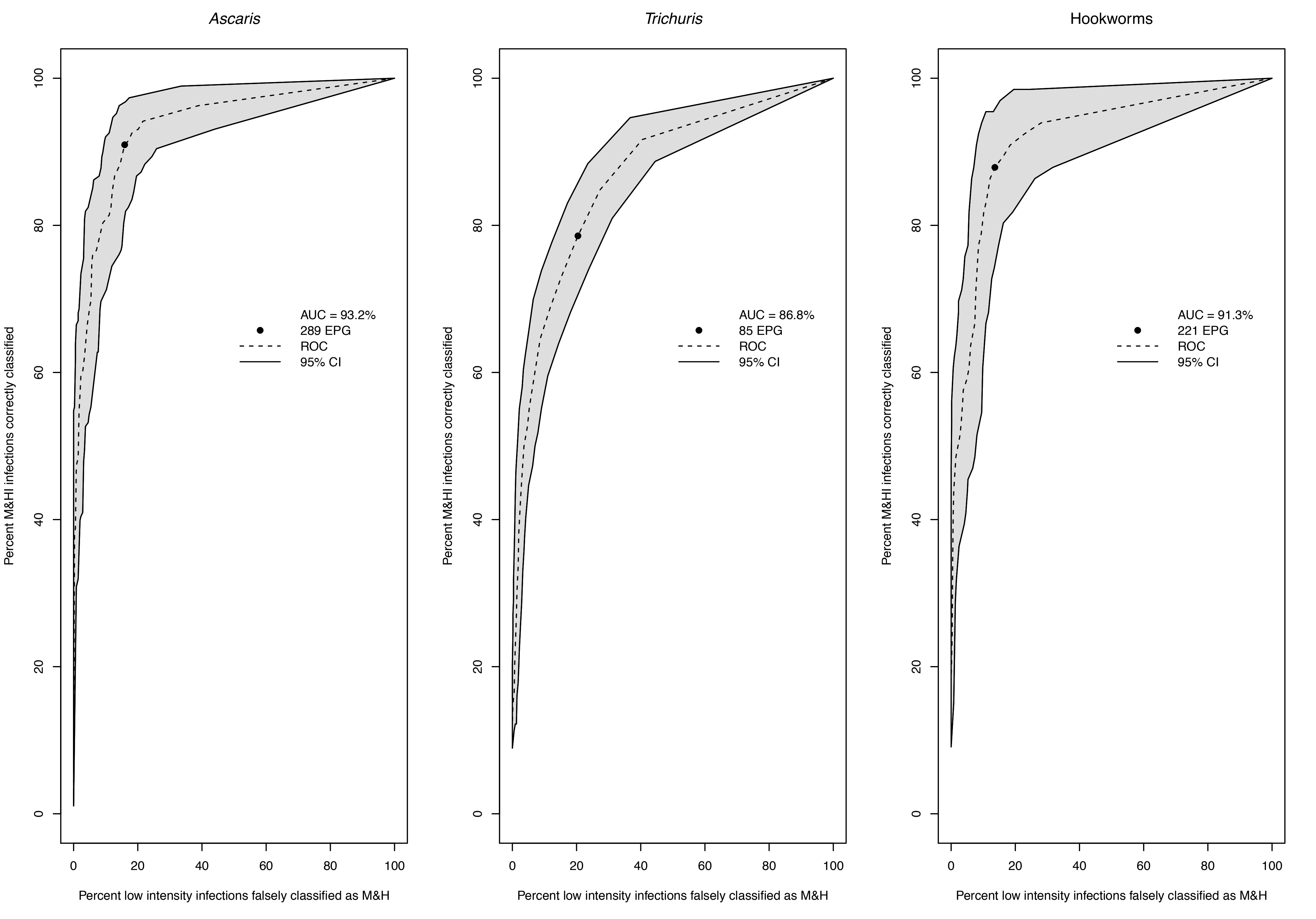
Ascaris
Trichuris
Hookworms
Percent M&HI infections correctly classified
Percent low intensity infections falsely classified as M&H
AUC = 93.2%
289 EPG
AUC = 86.8%
85 EPG
AUC = 91.3%
221 EPG
ROC
95% CI
0
20
40
60
80
100
