# Supplementary material for: Identifying thresholds for classifying moderate-to-heavy soil-transmitted helminth intensity infections for FECPAKG2, McMaster, Mini-FLOTAC and qPCR
Source: PLoS Negl Trop Dis. 2020 Jul 2;14(7):e0008296. doi: 10.1371/journal.pntd.0008296 (PMC7413557; doi:10.1371/journal.pntd.0008296)
Supplement: S1 Table — The table reports both the uncorrected and corrected prevalence of moderate-to-heavy (M&H) intensity infections for Mini-FLOTAC, FECPAKG2, McMaster and qPCR. The uncorrected prevalence equals to the number of samples classified as M&H intensity based on the method-specific thresholds (Table 4). The corrected prevalence and the corresponding 95% confidence intervals (95% CI) are based on the formulae described by Diggle (2011) [33]. (DOCX) [file pntd.0008296.s004.docx]

|  | | **Uncorrected prevalence of M&HI infections when applying method-specific thresholds (95%CI)** | **Proportion (%) of M&HI infections correctly classified** | **Proportion (%) of light intensity infections falsely classified as M&H** | **Corrected prevalence M&HI infections**  **(95% CI)** | **Prevalence M&HI infections based on a single Kato-Katz and WHO thresholds** |
| --- | --- | --- | --- | --- | --- | --- |
| ***Ascaris* (n_study 1_: 540; n_study 2_: 323)** | | | | |  |  |
|  | Mini-FLOTAC | 38.1 (34.0; 42.2) | 94.7 | 8.0 | 34.7 (30.0; 39.4) | 34.8 |
|  | FECPAK^G2^ | 42.0 (37.8; 46.2) | 91.0 | 15.9 | 34.8 (29.2; 40.3) | 34.8 |
|  | McMaster | 49.5 (44.0; 55.0) | 90.2 | 21.1 | 41.1 (33.2; 49.0) | 41.2 |
|  | qPCR | 37.0 (32.9; 41.1) | 83.5 | 12.2 | 34.8 (29.1; 40.5) | 34.8 |
|  |  |  |  |  |  |  |
| ***Trichuris* (n_study 1_: 889; n_study 2_: 354)** | | | | |  |  |
|  | Mini-FLOTAC | 41.2 (38.0; 44.4) | 93.5 | 9.4 | 37.8 (34.0; 41.7) | 37.8 |
|  | FECPAK^G2^ | 42.2 (39.0; 45.2) | 78.6 | 20.4 | 37.5 (31.9; 43.0) | 37.8 |
|  | McMaster | 33.3 (28.4; 38.2) | 82.1 | 24.2 | 15.7 (7.2; 24.2) | 15.8 |
|  | qPCR | 48.5 (45.2; 51.8) | 87.2 | 25.0 | 37.8 (32.5; 43.1) | 37.8 |
|  |  |  |  |  |  |  |
| **Hookworms (n_study 1_: 675; n_study 2_: 313)** | | | | |  |  |
|  | Mini-FLOTAC | 16.9 (14.1; 19.7) | 87.9 | 9.2 | 9.8 (6.2; 13.4) | 9.8 |
|  | FECPAK^G2^ | 20.9 (17.8; 24.0) | 87.9 | 13.6 | 9.8 (5.7; 14.0) | 9.8 |
|  | McMaster | 25.2 (20.4; 30.0) | 79.2 | 20.8 | 7.5 (0; 15.8) | 7.7 |
|  | qPCR | 18.1 (15.2; 21.0) | 78.8 | 11.5 | 9.8 (5.5; 14.1) | 9.8 |

**Supplementary Info S1 Table. The uncorrected and corrected prevalence of moderate-to-heavy intensity infections based on method-specific thresholds.** The table reports both the uncorrected and corrected prevalence of moderate-to-heavy (M&H) intensity infections for Mini-FLOTAC, FECPAK^G2^, McMaster and qPCR. The uncorrected prevalence equals to the number of samples classified as M&H intensity based on the method-specific thresholds (**Table 4**). The corrected prevalence and the corresponding 95% confidence intervals (95% CI) are based on the formulae described by Diggle (2011) [1].

**Reference**

1. Diggle PJ. Estimating prevalence using an imperfect test. Epidemiol Res Int. 2010; 608719.
